# Multiple adaptive and non-adaptive processes determine responsiveness to heterospecific alarm calls in African savannah herbivores

**DOI:** 10.1098/rspb.2017.2676

**Published:** 2018-07-04

**Authors:** Kristine Meise, Daniel W. Franks, Jakob Bro-Jørgensen

**Affiliations:** 1 Mammalian Behaviour and Evolution, Institute of Integrative Biology, University of Liverpool, Liverpool, UK; 2 Department of Biology, University of York, York, UK; 3 Department of Computer Science, University of York, York, UK

**Keywords:** interspecific communication network, alarm calls, adaptive response, mixed-species groups, herbivores

## Abstract

Heterospecific alarm calls may provide crucial survival benefits shaping animal behaviour. Multispecies studies can disentangle the relative importance of the various processes determining these benefits, but previous studies have included too few species for alternative hypotheses to be tested quantitatively in a comprehensive analysis. In a community-wide study of African savannah herbivores, we here, for the first time to our knowledge, partition alarm responses according to distinct aspects of the signaller–receiver relationship and thereby uncover the impact of several concurrent adaptive and non-adaptive processes. Stronger responses were found to callers who were vulnerable to similar predators and who were more consistent in denoting the presence of predators of the receiver. Moreover, alarm calls resembling those of conspecifics elicited stronger responses, pointing to sensory constraints, and increased responsiveness to more abundant callers indicated a role of learning. Finally, responses were stronger in risky environments. Our findings suggest that mammals can respond adaptively to variation in the information provided by heterospecific callers but within the constraints imposed by a sensory bias towards conspecific calls and reduced learning of less familiar calls. The study thereby provides new insights central to understanding the ecological consequences of interspecific communication networks in natural communities.

## 1. Introduction

Most studies investigating the role of communication in animal behaviour have focused on single-species groups [1,2]. However, there is increasing interest in information transfer between species and its role in shaping behaviours of animals living in mixed-species groups [3–5]. In particular, communication between species about predation risk often may have substantial fitness consequences by increasing survival chances during an attack [6,7]. Although interspecific communication benefits can be fundamentally important for social dynamics between species [8–10], the principles underlying behavioural responses to heterospecific informants remain poorly understood.

The value of heterospecifics as informants depends on their ability to detect a predator, their likelihood of emitting an alarm call upon detection and the extent to which they are vulnerable to the same predators as the receiver, i.e. the predator overlap [1,10]. Where the predator overlap is only partial, the reliability of heterospecific alarm calls may be reduced by ‘false positives’ (i.e. erroneously indicating a predator when none is present from the perspective of the receiver), whereas the consistency of a heterospecific alarm caller in denoting predator presence may be reduced by ‘false negatives’ (i.e. not indicating the presence of a predator from the perspective of the receiver) [2,7]. Accordingly, significant differences can be expected in the survival benefits that a species gains by responding to alarm calls of different heterospecifics.

But are animals able to respond adaptively to these differences in the information provided by heterospecific alarm calling? Some studies have indeed found alarm responses to depend on predator overlap [11,12], call reliability and caller consistency [13–16]. Still, other studies indicate that responses are also influenced by the similarity of the acoustic structure to the conspecific alarms [17,18], suggesting that sensory bias limits the ability to extract information from heterospecific alarm calls. Yet, other studies have found a positive correlation between responses to heterospecific alarm calls and familiarity with the calling species [19–21], indicative of learning. These hypotheses are not mutually exclusive, and responses to heterospecific alarm calls may well be the result of several factors operating simultaneously [6]. However, the limited number of species included in previous studies of interspecific alarm communication has precluded simultaneous statistical assessment of the various explanations proposed.

The alarm communication network of African savannah herbivores is an ideal system in which to study the relative importance of the factors purported to influence interspecific communication. In this system, multiple species are commonly found in mixed-species groups [22,23], and heterospecifics therefore have the potential to act as an important source of information about predation risk. Moreover, the species-rich guild provides pronounced diversity in key ecological variables, such as morphology, predator vulnerability and species abundance [24–26], and extensive variation can therefore be expected in the information content of heterospecific alarms and the associated detection benefits.

In the present study, we first establish the information content of the alarm calls of each herbivore species by identifying which predators trigger them. This allows us to assess to what extent species-specific alarms reflect the vulnerability to predators (table 1, H1). Next, we investigate the various adaptive and non-adaptive hypotheses proposed to explain the function of interspecific communication networks (table 1). Specifically, we test whether herbivores respond more strongly to alarm calls from species with whom predator overlap is high (H2), alarms calls from species who are more consistent in indicating when predators of the receiver are present (H3.1), alarm calls which more reliably indicate a predator to which the receiver is vulnerable (H3.2), more familiar alarm calls (H4) and alarm calls acoustically similar to those of the receiver (H5). Additionally, we test if responsiveness to alarm calls depends on environmental factors related to predation risk (H6). The species-rich study system allows us for the first time, to our knowledge, to quantitatively test the impact of interspecific relationships on alarm responses and thereby to gain new insights into the adaptive value of heterospecific alarm communication networks.

**Table 1.** Hypothetical framework.

hypothesis	predictions	references
H1: the information content of an alarm call reflects the predator vulnerability of the caller (adaptive)	species are more likely to give alarm calls in response to predators to which they are more vulnerable	[27]
H2: herbivores respond more strongly to alarm calls from species with similar predators (adaptive)	responsiveness is higher to alarm calls from species with body sizes similar to the receiver (proxy measure of predator overlap, [28,29])	[11,12]
H3: receivers respond more strongly to more accurate information sources (adaptive) H3.1: receivers respond more strongly to alarm calls from more consistent informants H3.2: receivers respond more strongly to more reliable alarm calls	3.1: responsiveness is higher to alarm calls from species emitting few false negatives 3.2: responsiveness is higher to alarm calls from species emitting few false positives	[13–16]
H4: receiver responses are influenced by learning (adaptive, but limited to more familiar calls)	responsiveness is higher to calls from more abundant heterospecifics	[19–21]
H5: receivers are more sensitive to calls similar to their own (non-adaptive)	responsiveness is higher to alarm calls which are acoustically similar to the conspecific alarms	[17,18]
H6: receiver responses are influenced by environmental factors affecting predation risk (adaptive and non-adaptive)	responsiveness increases with grass height responsiveness decreases with proximity to cover responsiveness increases with wind speed responsiveness decreases with distance to caller responsiveness decreases with group size	[28]

## 2. Material and methods

### (a) Study system

The study was conducted between September 2015 and October 2016 in the Masai Mara National Reserve, Kenya, which is part of the Serengeti-Mara Ecosystem and characterized by open savannah grassland and riverine forests. We focused on the 12 most common species in the herbivore community: Thomson gazelle (*Gazella thomsonii*, ‘Tho’), Grant gazelle (*Gazella granti*, ‘Gra’), impala (*Aepyceros melampus*, *‘*Imp’), common warthog (*Phacochoerus aethiopicus*, ‘War’), ostrich (*Struthio camelus*, ‘Ost’), topi (*Damaliscus lunatus*, ‘Top’), hartebeest (*Alcelaphus buselaphus*, ‘Har’), blue wildebeest (*Connochaetes taurinus*, ‘Wil’), plains zebra (*Equus quagga*, ‘Zeb’), African buffalo (*Syncerus caffer*, ‘Buf’), common eland (*Tragelaphus oryx*, ‘Ela’), and giraffe (*Giraffa camelopardalis*, ‘Gir’). Their main predators include the lion (*Panthera leo*), spotted hyena (*Crocuta crocuta*), leopard (*Panthera pardus*), cheetah (*Acinonyx jubatus*), and black-backed jackal (*Canis mesomelas*).

### (b) Ecological and morphological species characteristics

To calculate the relative abundance of the study species, we conducted a total of 66 censuses at approximately 16 day intervals on three study plains, covering a total of 54 km^2^. We then determined relative abundance of the study species from the mean number of individuals recorded per census. We used abundance data of all predator species collected by Broekhuis [30] during transects to calculate relative predator abundance. Vulnerability to predators was quantified using the Jacob's index [31–35] (transformed to values between 0 and 1, with values close to 1 indicating a high vulnerability to predators). As no indices were given for the preference of the black-backed jackal for Thomson and Grant gazelles, we used the value reported for the closely related springbok (*Antidorcas marsupialis*) which is similar in size, speed and ecological niche. The body size ratio between caller and receiver was calculated based on the mean adult body mass [24,36]. Following Lovich & Gibbons [37], we calculated the body size ratio as [receiver mass : caller mass] when the receiver was larger, and [2 − (caller mass : receiver mass)] when the receiver was smaller than the caller.

### (c) Call reliability and caller consistency

To determine the probability with which species-specific alarm calls denoted the various predators (i.e. their information content), we conducted a predator simulation experiment where we exposed the study species to life-sized lateral photographs of their five main predators (see ‘Study system’) and a reedbuck (*Redunca redunca*) as a control. The two-dimensional models were presented to monospecific groups (for details on the experimental design, see the electronic supplementary material, S2). Once the first animal in the group detected the model (i.e. looked straight at the model with pointed ears), we noted the occurrence of alarm calls emitted over the next 5 min. We determined the distance to the model (using a laser range finder, Bushnell Scout DX 1000 ARC), group size and the presence of young individuals (i.e. less than half the adults' body shoulder height). In total, we conducted 649 predator simulations aiming for an even distribution of simulations between the predator–herbivore combinations (mean ± s.e. = 9.05 ± 0.26).

To identify the relative importance of false-negative and false-positive alarm calls in the interspecific communication, we distinguished the value of an alarm caller from the value of a single alarm call as information sources. Hence, we differentiated: (i) the consistency of an alarm caller in denoting the presence of the receiver's predators whenever these are present; and (ii) the reliability of a single alarm call in indicating a predator to which the receiver is vulnerable. The caller consistency was calculated as the probability that an alarm call is emitted when the signaller is presented with a given predator model, weighted by the relative probability of encountering that predator, multiplied by the vulnerability of the receiver to that predator, summed over all predators in the system:
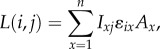
where *i* denotes the species identity (ID) of the receiver; *j* the caller species ID; *n* the number of predator species; *I_xj_* the probability that species *j* gives an alarm call in response to a model of predator *x*; *ɛ_ix_* the preference of predator *x* for species *i*; and *A_x_* the relative abundance of predator *x*. A high value of *L*(*i*, *j*) (close to 1) suggests that species *j* is highly likely to inform about the presence of species *i*'s predators.

Following Magrath *et al*. [13], we calculated the reliability of a species' alarm call as
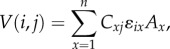
where *C_xj_* denotes the proportion of alarm calls of species *j* elicited by the model of predator *x* when models of all predators are presented with equal frequency. A high value of *V*(*i*, *j*) (close to 1) indicates that an alarm call of species *j* is likely to be directed to a predator to whom species *i* is highly vulnerable.

Note that we thus distinguish callers and calls as being more or less consistent, respectively, reliable (a continuous approach) rather than as being true or false (a categorical approach).

### (d) Acoustic structure of alarm calls

Alarm calls were collected ad libitum during natural predator–prey encounters observed during previous fieldwork in the study area (2011–2016) using a digital audio recorder (Marantz PMD670) with a directional microphone (Sennheiser ME67). Given the stereotypic acoustic structure of alarm calls within each species, we combined all the alarm calls according to species for further analysis (see the electronic supplementary material, S2 for details). We analysed 10 high-quality calls from different individuals of each study species except the ostrich (*n* = 9) and the eland (*n* = 0; alarm calls were never heard during previous long-term fieldwork on the species in the study area and therefore considered unimportant, [38]). The acoustic similarity between alarm calls was quantified as (1 − Euclidean distance) using the following variables: duration, visibility of harmonics, number of distinct structural components, presence of pulses, the 25% energy quartile, the bandwidth between the 25% and the 75% energy quartiles and the third dominant frequency, DF3 (because DF1, DF2 and DF3 were highly correlated, we only included DF3 which showed most interspecific variation and best separated species; for details on the acoustic analysis, see the electronic supplementary material, S2). Each measure was standardized by dividing each value by the maximum value of this measure to ensure equal weighting of variables.

### (e) Alarm responses

For the playback experiment, we selected six high-quality recordings from each of the 11 vocal study species, three from each sex. As a control, we used three recordings of a non-alarm call from the ring-necked dove (*Streptopelia capicola*), which is frequently heard throughout the study area. Using a digital sound level meter (UNI-T, model UT352), we determined species-specific alarm call intensity at 35 m distance in the wild, and subsequently, we adjusted playback volume to natural levels by matching sound level meter measurements at this distance, where average intensity for the study species ranged from 54 to 67 dB.

We conducted a total of 2433 playback experiments following a balanced design in terms of the species and sex of both caller and receiver (for each caller–receiver combination: mean ± s.e. = 17.7 ± 0.43). The playback experiments were targeted at animals which were relaxed and foraging for at least 20 s prior to the experiment, and the response was recorded using a digital video camera (Sony HDR-PJ810E). For each experiment, we recorded wind speed (using an anemometer, Proster Digital LCD), distance of the focal animal (using the laser rangefinder), group size and estimates of grass height and proximity to cover (for details on the playback design, see the electronic supplementary material, S2).

We analysed the playback videos using BORIS (Behavioural Observation Research Interface Software, [39]). Responses were coded both as a binary variable, where a response was defined as any behavioural change taking place within 10 s after the playback sound, and as a continuous variable, where response strength was measured by the latency to first response, speed of head-lifting, time until foraging was resumed for at least 10 s and number of head-ups and scratches (electronic supplementary material, S1).

### (f) Statistical analysis

All analyses were performed in R v. 3.4.0 [40]. Model selection was based on the Akaike information criterion for small sample sizes (AICc) (*MuMIn* package, [41]; for full model descriptions, see the electronic supplementary material, S3 and S4). Results presented refer to the models with the lowest AIC. *P*-values for mixed models were obtained using the Kenward–Rogers method for linear mixed models and likelihood ratio tests for generalized linear mixed models (*afex* package, [42]). Integer variables were standardized by mean centring and scaling by the standard deviation. Final models were checked for overdispersion and multicollinearity. For linear models, we additionally checked normality and homoscedasticity of residuals. For three variables, the assumption of normality was violated, but after log-transforming the response variable all model assumptions were met.

To assess the information content of alarm calls (H1), we modelled the probability of giving an alarm call as a function of predator identity using logistic regression (*lme4* package, [43]). Initially, we included focal species ID, model type (predator/control) and their interaction term as explanatory variables. This confirmed that all species had a higher probability of giving an alarm call when presented with a predator model compared with the control (*n* = 626 experiments; *b* = 1.35, *z* = 4.61, *p* < 0.001). We subsequently tested the effect of species-specific predator vulnerabilities on the probability of alarm calling to the five predator models, including focal species ID, predator vulnerability, their interaction, distance to the model, group size and the presence of young as explanatory variables (M1, *n* = 522 experiments).

To determine species-specific differences in alarm responses, we modelled response probability as the binary response variable in a logistic regression model with receiver species ID, call type (conspecific/heterospecific/control), their interaction, grass height, proximity to cover, distance to speaker, wind speed and group size as explanatory variables (*n* = 2433 experiments). As the response probability differed significantly between control and alarm sounds (conspecific call: *b* = 3.20, *z* = 10.00, *p* < 0.001; heterospecific call: *b* = 2.37, *z* = 9.62, *p* < 0.001) and individuals were no more likely to raise their heads during control playbacks than during undisturbed foraging bouts (Wilcoxon signed-rank test: *V* = 55, *p* = 0.117), we removed the control sound from further analyses, replacing call type with caller species ID (M2, *n* = 2334 experiments).

To assess the adaptive value of alarm calls (H2–H6), we analysed the probability to respond to heterospecific alarm calls using a binomial mixed effect model with logit-link function with the following explanatory variables: receiver's body size, body size ratio (including linear and quadratic terms as we expected the highest responsiveness to callers of the same size), the interaction between the receiver's body size and the body size ratio (linear and quadratic term), caller consistency, call reliability, acoustic similarity and abundance of the caller. Additionally, we included grass height, proximity to cover, distance to speaker, wind speed and group size (M2.1, *n* = 2030 experiments); receiver species ID was included as a random factor. Response strength was analysed using separate log-linear mixed models for latency (M2.2, *n* = 1529 experiments), duration (M2.3, *n* = 1429 experiments) and speed of head-lifting (M2.4, *n* = 1466 experiments), and generalized linear mixed effect models with negative binomial distribution for the number of head-ups and scratches (M2.5 and M2.6, *n* = 1380 experiments); the explanatory variables and the random factor were the same as in the previous model.

## 3. Results

### (a) Do information content of alarm calls and receiver responses differ between species?

**Figure 1.**
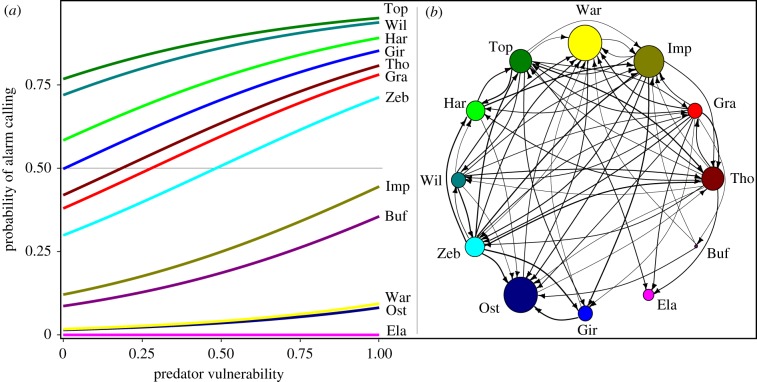
Communication network of African savannah herbivores. (*a*) Species-specific differences in the probability of alarm calling in relation to predator vulnerability. (*b*) Species-specific dependency on heterospecific alarm calls. Arrows point to species in which alarm calls elicited a response with edge weight representing response probability (cut-off point: 0.72). Node-size indicates the number of species whose alarm calls caused a response (for species abbreviations, see ‘Study system’). (Online version in colour.)

The study species differed in their general probability of alarm calling when exposed to a predator model (M1, 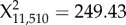, *p* < 0.001; figure 1*a*), and the probability that a species would alarm call to a given predator model depended on its vulnerability to that predator (*b* = 1.76, *z* = 3.89, *p* < 0.001) (H1). This indicates that both the consistency of the caller and the reliability of the alarm calls differ significantly between species that vary in predator overlap. In line with this finding, individual species showed pronounced asymmetries in their probability of responding to alarm calls from different species (M2, 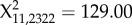, *p* < 0.001), leading to a directed communication network among savannah herbivore species (figure 1*b*). Individuals were generally more responsive to conspecific alarm calls than to heterospecific alarm calls (*b* = 0.96, *z* = 4.15, *p* < 0.001).

### (b) Are responses to heterospecific alarm calls adaptive or non-adaptive?

**Figure 2.**
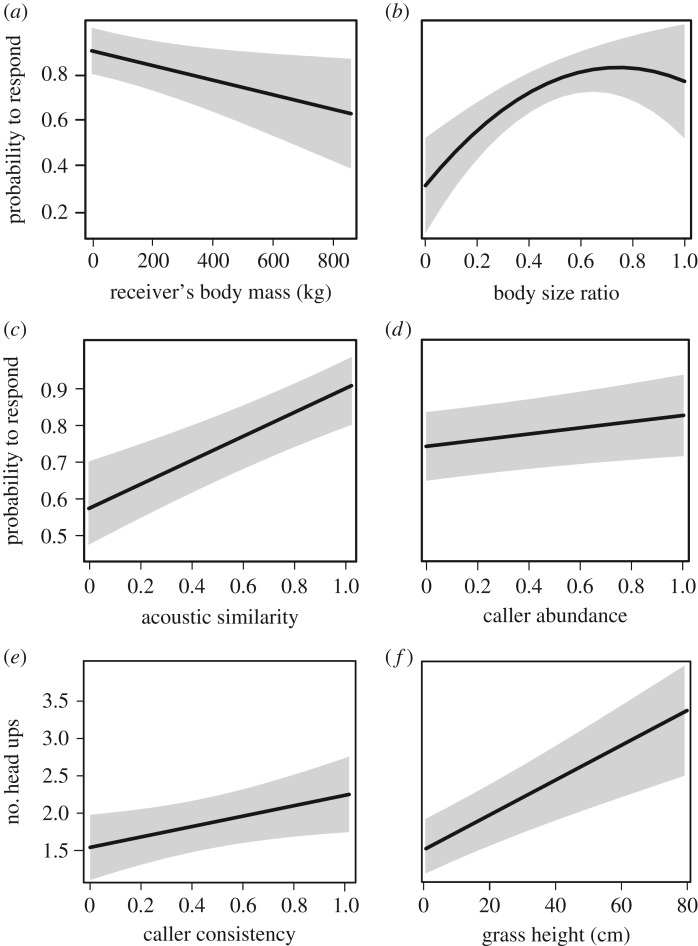
Probability of responding to an alarm call in relation to the body size of the receiver, the body size ratio between the caller and the receiver (H2), the acoustic similarity between caller and receiver alarms (H5) and the abundance of the caller (H4) (*a–d*). Head-up response to alarm calls in relation to the consistency of the caller (H3.1) and grass height (H6) (*e*,*f*). Body size ratio, acoustic similarity, abundance and consistency of the caller were all scaled between 0 and 1.

Responsiveness was highest towards alarm calls of similar-sized and slightly larger heterospecifics (response probability (M2.1), latency (M2.2), duration (M2.3) and scratches (M2.6); table 2 and figure 2*b*), indicating that herbivore species with similar predators are more likely to react to each other's alarm calls (H2). Moreover, larger species were generally less responsive (response probability (M2.1), latency (M2.2), speed of head-lift (M2.4) and scratches (M2.6); table 2 and figure 2*a*), and the significant interaction between receiver's body size and the body size ratio indicates that they are less sensitive to body size differences between the caller and the receiver (duration (M2.3) and scratches (M2.6); table 2).

**Table 2.** Responsiveness to heterospecific alarm calls in the savannah herbivore community.

model	response variable	statistics	explanatory variables											
			H2					H3.1	H3.2	H4	H5	H6		

			receiver body size (RBS)	body size ratio	body size ratio^2^	RBS : size ratio	RBS : size ratio^2^	caller consistency	call reliability	caller abundance	acoustic similarity	grass height	distance to caller	wind speed
M2.1	response probability	*b*	−0.45	6.48	−4.35					0.44	1.74	0.37	−0.08	0.09
		*X*^2^	4.56	16.61	7.64					5.57	31.94	31.47	2.04	2.82
		*p*-value	<0.05	<0.001	<0.01					<0.05	<0.001	<0.001	n.s.	n.s.
M2.2	latency	*b*	0.31	−2.81	1.40						−0.42		0.10	−0.07
		*F*	10.98	11.91	3.12						9.25		13.84	9.06
		*p*-value	<0.01	<0.001	n.s.						<0.01		<0.001	<0.01
M2.3	duration	*b*	−1.03	2.74	0.67	3.38	−3.07			0.21	0.56	0.12		0.12
		*F*	2.23	1.75	0.08	3.18	5.68			5.02	10.65	14.58		16.21
		*p*-value	0.14	n.s.	n.s.	n.s.	<0.05			<0.05	<0.001	<0.001		<0.001
M2.4	speed of head-lift	*b*	0.22											
		*F*	9.43											
		*p*-value	0.01											
M2.5	head-ups (number)	*b*	−0.11					0.26	−0.28			0.12		
		*F*	3.03					5.58	0.26			30.31		
		*p*-value	n.s.					<0.05	n.s.			<0.001		
M2.6	scratches (number)	*b*	1.22	1.47		−3.28						0.19		
		*F*	7.63	5.71		3.22						7.86		
		*p*-value	<0.01	<0.05		n.s.						<0.01		

Responsiveness was furthermore higher to alarm calls from those heterospecifics who were more consistent as informants (head-ups (M2.5); table 2 and figure 2*e*), suggesting that receivers are sensitive to false negatives (H3.1). We found no independent effect of the reliability of the alarm call itself (M2.1–M2.6, table 2), suggesting that any effect of emitting false positives was negligible (H3.2).

Responsiveness, moreover, increased with the abundance of the caller species (response probability (M2.1) and duration (M2.3); table 2 and figure 2*c*), suggesting that alarm responses are enhanced by learning (H4). In addition, responsiveness increased with similarity in the acoustic structure of the call to the receiver's own alarm call (response probability (M2.1), latency (M2.2) and duration (M2.3); table 2 and figure 2*d*), indicating that sensory constraints affect alarm responses (H5).

Finally, responsiveness increased with grass height (response probability (M2.1), duration (M2.3), head-ups (M2.5) and scratches (M2.6); figure 2*f*), wind speed (response probability (M2.1), latency (M2.2) and duration (M2.3)) and proximity to the caller (latency (M2.2)), whereas no significant effects were found of proximity to cover, or group size (electronic supplementary material, S4). These results support that the environmental context can affect alarm responses (H6).

These findings show that the responses of African savannah herbivores to heterospecific alarm calls are shaped by a range of factors which are partly adaptive, as indicated by the effects of body size similarity, caller consistency and grass height which affects predation risk, but also partly non-adaptive, notably depending on the acoustic similarity between the conspecific and heterospecific calls.

## 4. Discussion

Prey species often obtain information about the presence of predators from heterospecific alarm calls. Although this use of public information is widespread, we still know little about how individuals process other species' alarm calls [7]. In the present study, we established the information content of alarm calls from the community of African savannah herbivores and then quantified species-specific alarm responses in order to test the relative importance of different adaptive and non-adaptive processes. Our results indicate that responses to heterospecific calls increase with the predator overlap between the caller and the receiver, the consistency of the caller from the perspective of the receiver and the predation risk in the environment, suggesting that part of the response to heterospecific alarm calls is adaptive. However, we also found an independent effect of acoustic similarity, which indicates that perception is limited by sensory constraints. These findings reveal that the alarm communication network of savannah herbivores is the outcome of multiple forces acting simultaneously.

Both predation and resource limitation are crucial factors in regulating the herbivore populations of the African savannah [44], and a primary expectation of our study was therefore that the study species are optimizing the trade-off between benefits from increased predator detection and costs from reduced foraging in their responsiveness to heterospecific alarm calls [27,45]. In particular, strong selection was expected to favour increased responsiveness to species sharing similar predators. We indeed found that receivers respond more strongly to alarm calls from similar-sized or slightly larger species with whom predator overlap is high (H2, table 1). Receivers may therefore use an awareness of similarity in predator vulnerability to assess the importance of alarm calls from heterospecifics.

In this study, we moreover distinguished the reliability of a single alarm call in denoting a predator of the receiver from the consistency of the heterospecific caller in denoting when a predator of the receiver was present. In doing so, we identified an effect of the consistency of the caller (i.e. few false negatives, H3.1), but not the reliability of the alarm call (i.e. few false positives, H3.2). This suggests that it is more important that a heterospecific consistently gives alarm calls when encountering a predator of the receiver than whether the heterospecific also gives irrelevant alarm calls to carnivores which are not predators of the receiver. It is possible that the consistency in hearing a given heterospecific calling whenever a predator is encountered facilitates learning of the information content of the alarm call. This explanation is supported by the increased responsiveness to alarm calls from more abundant species: learning of their alarm calls is likely to be facilitated by hearing them more frequently (H4). An effect of learning is consistent with the conclusion of a previous study of fairy-wrens (*Malurus cyaneus*) in which the fact that heterospecific alarms only elicited alarm responses in sympatry, and not in allopatry, was interpreted as demonstrating a role of learning [20,46,47]. While this single-species study was also able to conclude that call similarity was ‘neither sufficient nor necessary for interspecific recognition’ (p. 769), our multispecies study demonstrates that there is still an additional effect of acoustic similarity on alarm responses at the community level (H5). This is consistent with other studies which have reported unlearned responses to acoustically similar heterospecific calls where responses to conspecific alarm calls are innate [12,17,48]. Hence, our study suggests that although both awareness of the social environment and associative learning of acoustic signals shape alarm responses, sensory bias limits the flexibility in responding adaptively to heterospecifics calls depending on their similarity to that of conspecifics. Further studies are needed to fully understand the underlying cognitive processes.

Our findings also suggest that herbivores adjust their alarm responses to environmental factors increasing predation risk [28] (H6). Stronger responses were found to alarm calls when heard on plains with longer grass. This is probably an adaptive precaution because stalking predators are dependent on cover provided by the grass to get sufficiently close to their prey to launch a successful attack on open plains [29]. It is also conceivable that enhanced food abundance on long grass swards diminishes the costs from foraging foregone when responding to alarms. Alarm calls moreover elicited stronger responses when heard from a closer distance, again suggesting adaptive adjustment to heightened predation risk. Finally, stronger responses under windy conditions can likewise be explained as an adaptation to increased risk of predation [49]. Ungulates are known to increase group size and seek safe habitats as antipredator precautions under windy conditions where their ability to detect predators decreases [50]. Although we only played alarm calls at wind speeds that assured their detection by the intended receiver, distortion of transmission may still have impeded the localization of predators by acoustic and olfactory cues at the higher wind speeds below this threshold.

The array of factors demonstrated to simultaneously influence the responses to heterospecific alarm calls in this study highlights the importance of multivariate analysis at the species level in deciphering interspecific alarm communication networks. Insights into the relative importance of the crucial factors in turn deepen our understanding of the social landscape in which interspecific interactions unfold. In particular, the role of communication as a driver of social affinity between species and the formation of mixed-species groups requires an in-depth understanding of both the information content encoded in alarm calls and how this information is decoded by the receiver. We have here shown that alarm responses of savannah herbivores are only partly adaptive and that an appreciation for limitations to adaptation is likely to be critical for understanding the role of interspecific communication in shaping ecological processes.

## Supplementary Material

S1

## Supplementary Material

S2

## Supplementary Material

S3

## Supplementary Material

S4

## Data accessibility

Data are available from the Dryad Digital Repository: http://dx.doi.org/10.5061/dryad.mb7dd20 [51].
