# Supplementary material for: Multiple adaptive and non-adaptive processes determine responsiveness to heterospecific alarm calls in African savannah herbivores
Source: Proc Biol Sci. 2018 Jul 4;285(1882):20172676. doi: 10.1098/rspb.2017.2676 (PMC6053937; doi:10.1098/rspb.2017.2676)
Supplement: S1 [file rspb20172676supp1.docx]

S1: Definition of the behaviours categorized in response to alarm calls.

| **Behaviour** | **Description** |
| --- | --- |
| **Response [yes/no]** | A response was defined as any behavioural change taking place within 10 seconds after the playback sound. This ranges from subtle responses, such as no visible mouth movements for a few seconds, over intermediate responses where individuals lifted their head, to strong responses when individuals ran off. To quantify differences in response strength we focused on the variables described below. |
| **Latency [ms]** | Time until the receiver stopped foraging. |
| **Duration [ms]** | Time from when the receiver stopped foraging to when foraging was resumed for at least 10 seconds without interruption. |
| **Speed of head-lift [ms]** | Speed with which an animal lifted its head. Measured from when it started lifting the head to when the head was fully raised (i.e. above shoulder level or, for the buffalo, at shoulder level). |
| **Head-ups** | Number of times the head was lifted to shoulder level or, for buffalo and wildebeest, slightly below before foraging was resumed for at least 10 seconds. |
| **Scratches** | Number of scratching bouts before foraging was resumed for at least 10 seconds. |
