# Supplementary material for: Multiple adaptive and non-adaptive processes determine responsiveness to heterospecific alarm calls in African savannah herbivores
Source: Proc Biol Sci. 2018 Jul 4;285(1882):20172676. doi: 10.1098/rspb.2017.2676 (PMC6053937; doi:10.1098/rspb.2017.2676)
Supplement: S2 [file rspb20172676supp2.docx]

S2: Additional information on the experimental design

*Predator simulations*

For the predator experiments, we selected monospecific groups and observed them for at least one minute to assure that all animals were relaxed. Hidden from view of the focal group, the predator model was mounted on metal stands beside the car and revealed by slowly driving the car forward at least 25 meters. Once the first animal in the group detected the model (i.e. looked straight at the model with pointed ears), we noted the occurrence of alarm calls emitted over the next 5 minutes. Although we used a single photo model for each species and therefore pseudo-replicated at the level of predator model, we think it is unlikely that responses were specific to the particular model used, as the proportion of events which elicited alarm calls from one or more individuals of a specific prey species was highly correlated between the predator simulation experiments and 128 natural predator-prey encounters observed (*N* = 29 predator-prey combinations, Pearson correlation coefficient = 0.554, *p* = 0.002), confirming the suitability of predator models in determining the information content of alarm calls.

*Acoustic analysis*

Fourier-transformed spectrograms of calls were analysed in Praat 6.0.26 (settings: Gaussian window length: 0.025s, time steps: 1000, frequency steps: 2500; [1]). Each call was categorized as noisy or tonal depending on the visibility of harmonics, and we noted the number of distinct structural components in the time domain (figure 1*A*), and the presence/absence of multiple pulses (figure 1*B*); inter-observer reliability of these three measures was 100% (calculated for a subset of 5.5% of the alarm calls). We then recorded its duration and determined the dominant frequencies (i.e. the three bands with the highest frequency in the spectrum) from a spectrum after cepstral smoothing (1000Hz). We moreover described the distribution of energy in the call by determining the frequency quartiles below which 25% respectively 75% of the energy was found using the ‘SpectrumTier’ function: the difference between these quartiles then reflected the bandwidth of the call. Here, we first subtracted the energy of the background noise (measured before the onset of the alarm call) from the energy of the call to avoid an influence of background noise on the distribution of energy.


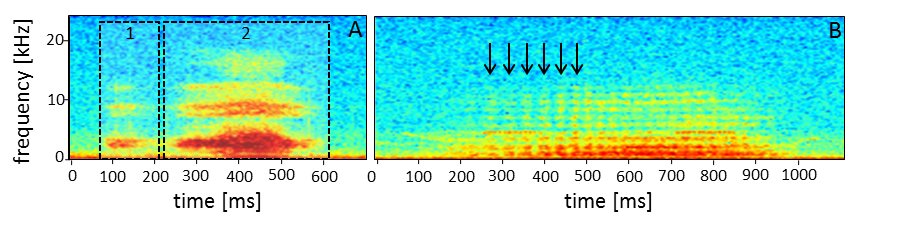


Figure 1: Spectrograms showing the two distinct components in the alarm calls of Thomson gazelles (A) and the visible pulses in the alarm calls of giraffes (B).

We conducted a principal component analysis of 6 acoustic variables describing the alarm calls and used the loadings of the first PC (which explained 87% of the variation) to analyse whether species differed in the acoustic structure of their alarm calls. Whereas differences between species in their alarm calls were detectable by the human ear, alarm calls to different predators did not generally appear to differ within species, and for a subset of species with an adequate number of alarm calls in response to different predator types, we tested the assumption that alarm calls were stereotypic within species. The acoustic structure of alarm calls was indeed found to differ significantly between species (*N* = 108, *F* = 79.772, *p* < 0.001, figure 2) and to be independent of the predator eliciting the alarm call (calculated for Grant gazelles, impalas and topis*: N* = 96, *F* = 1.102, *p* = 0.353). We therefore lumped all the alarm calls of each species and chose the highest quality recordings as playback stimuli.


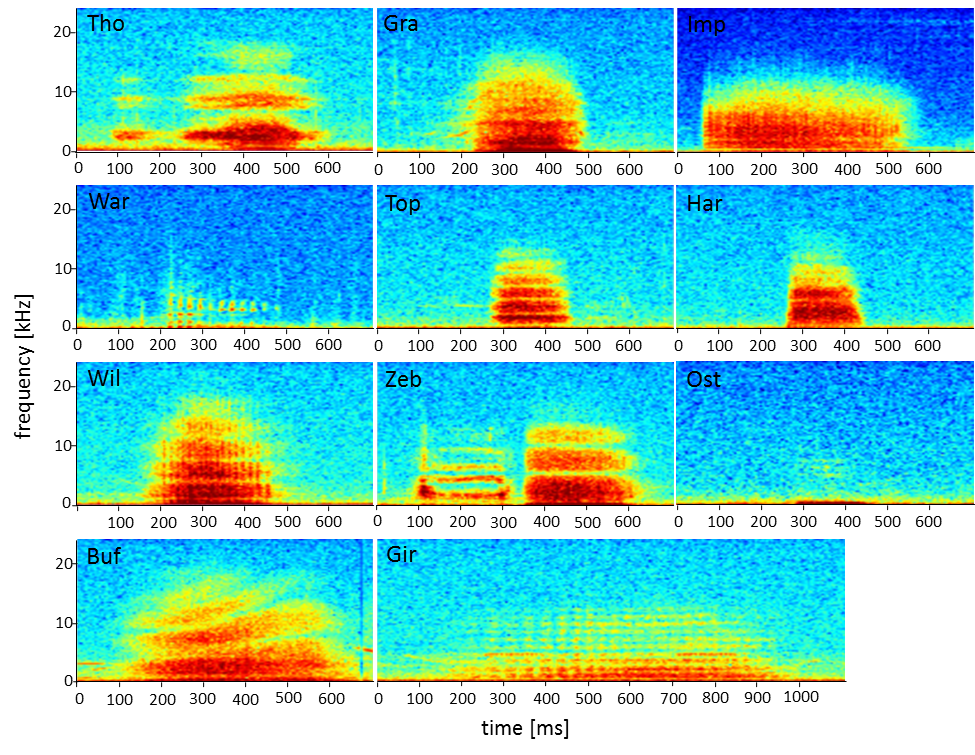
Figure 2: Spectrograms of alarm calls of the 11 vocal study species.

*Playback experiments*

We used a combination of short-term individual recognition and spatial and temporal separation to avoid an effect from pseudo-replication due to repeated testing of the same individuals. On a given day, we spaced experiments sufficiently far apart (minimum: 250m) to assure that any focal animal was out of earshot of previous experiments, and we left a minimum of three days between experiments in the same general area, each area covering approximately 20km^2^. Of the on average 96 playback experiments conducted for each sex of the 12 study species, only 13.2% were conducted in the same general area, and on average, a particular species was exposed to only 1.27 playbacks of a given call type within the same general area. The large size of the study area and the large population sizes of the study species [2] minimized the probability that the same individuals were tested repeatedly. To further avoid any issues due to effects of pseudo-replication, we moreover divided the year into five sampling intervals during which we tested males and females of each receiver species with a specific call type only once.

The playback experiments were targeted at foraging individuals with the animal closest to the car chosen as the focal animal. The loudspeaker (Mipro MA707, connected to Tascam HD-P2 audio recorder) was placed at a distance of an average of 65 meters and a 90⁰ degree angle to the animal, hidden by the silhouette of the car, and the playback stimulus was decided by a randomized spreadsheet. To avoid an impact of degradation of alarm calls on playback responses, we restricted playback distance to <100m (mean ± s.e. = 64 ± 0.29m) and we did not perform any experiments when the wind speed exceeded 6m/s (mean ± s.e. = 1.15 ± 0.02m/s).

[1] Boersma P, Weenink D. 2017 Praat: doing phonetics by computer [Computer program]. Version 6.0.30, <http://www.praat.org/>

[2] Ogutu J.O., Owen-Smith N., Piepho H.P., Said M.Y. 2011 Continuing wildlife population declines and range contraction in the Mara region of Kenya during 1977-2009. *J Zool* **285**(2), 99-109. (doi:10.1111/j.1469-7998.2011.00818.x).
