# Supplementary material for: Multiple adaptive and non-adaptive processes determine responsiveness to heterospecific alarm calls in African savannah herbivores
Source: Proc Biol Sci. 2018 Jul 4;285(1882):20172676. doi: 10.1098/rspb.2017.2676 (PMC6053937; doi:10.1098/rspb.2017.2676)
Supplement: S3 [file rspb20172676supp3.docx]

S3: Summary of models.

| **Question** | **No** | **Model** | **Subjects** | **Response variable** | **Explanatory variables** |
| --- | --- | --- | --- | --- | --- |
| Information content (H1) | M1 | Binom. GLM | All predator simulations | Call (yes/no) | Focal species * predator vulnerability ^a)^ |
| Species-specific alarm responses:  Adaptive or non-adaptive? (H2-H6) | M2 | Binom. GLM/ | All playback experiments | Response  (yes/no) | Receiver species * Caller species ^b)^ |
|  | M2.1 | Binom. GLMM | Heterospecific callers | Response  (yes/no) | Receiver body size * (Body size ratio + Body size ratio^2^) + Acoustic similarity + Caller consistency + Call reliability + Caller abundance ^b), c)^ |
|  | M2.2 | Log-LMM | Heterospecific callers | Latency | Receiver body size * (Body size ratio + Body size ratio^2^) + Acoustic similarity + Caller consistency + Call reliability + Caller abundance ^b), c)^ |
|  | M2.3 | Log-LMM | Heterospecific callers | Duration | Receiver body size * (Body size ratio + Body size ratio^2^) + Acoustic similarity + Caller consistency + Call reliability + Caller abundance ^b), c)^ |
|  | M2.4 | Log-LMM | Heterospecific callers | Speed of head-lift | Receiver body size * (Body size ratio + Body size ratio^2^) + Acoustic similarity + Caller consistency + Call reliability + Caller abundance ^b), c)^ |
|  | M2.5 | NB- GLMM | Heterospecific callers | Head-ups | Receiver body size * (Body size ratio + Body size ratio^2^) + Acoustic similarity + Caller consistency + Call reliability + Caller abundance ^b), c)^ |
|  | M2.6 | NB- GLMM | Heterospecific callers | Scratches | Receiver body size * (Body size ratio + Body size ratio^2^) + Acoustic similarity + Caller consistency + Call reliability + Caller abundance ^b), c)^ |

1. Distance to model + group size + presence of young
2. Grass height + proximity to cover + distance to speaker + wind speed + group size
3. Random effect: Receiver species
