# Supplementary material for: Multiple adaptive and non-adaptive processes determine responsiveness to heterospecific alarm calls in African savannah herbivores
Source: Proc Biol Sci. 2018 Jul 4;285(1882):20172676. doi: 10.1098/rspb.2017.2676 (PMC6053937; doi:10.1098/rspb.2017.2676)
Supplement: S4 [file rspb20172676supp4.docx]

S4: Model comparison based on AICc. Model fit is shown by delta AICc and model weight. For M2.1, M2.5 and M2.6, we only present models with a delta < 1.0. Grey indicates the models presented in the results.

| **NO** | **Response** | **Explanatory variables** | | | | | | | | | | | **Model details** | | | | | | | |  |
| --- | --- | --- | --- | --- | --- | --- | --- | --- | --- | --- | --- | --- | --- | --- | --- | --- | --- | --- | --- | --- | --- |
| M1 | Call | Species | Vulnerability | |  | Distance |  | Presence of young | | | |  | | df | | AICc | | delta | | weight | |
|  |  | + | + | |  |  |  | + | | | |  | | 14 | | 481.0 | | 0 | | 0.510 | |
|  |  | + | + | |  |  |  |  | | | |  | | 13 | | 482.8 | | 1.86 | | 0.201 | |
|  |  | + | + | |  | + |  | + | | | |  | | 15 | | 482.9 | | 1.90 | | 0.197 | |
| M2 | Res-  ponse | Receiver species | | Caller species | Distance | | | Grass |  | Wind | Group size | | df | | AICc | | delta | | weight | |  |
|  |  | + | | + | + | | | + |  |  |  | | 24 | | 2302.8 | | 0 | | 0.427 | |  |
|  |  | + | | + | + | | | + |  | + |  | | 25 | | 2302.8 | | 0.07 | | 0.413 | |  |
|  |  | + | | + | + | | | + |  |  | + | | 25 | | 2304.7 | | 1.96 | | 0.160 | |  |

| M  2.1 | Res-ponse | RBS^a)^ | Body size ratio | Body size ratio^2^ | RBS: size ratio | RBS: size ratio^2^ | Caller consis-tency | Call relia-bility | Acoustic similarity | Caller abun-dance | Grass height | Dis-tance | Wind  speed | group size | df | AICc | delta | weight |
| --- | --- | --- | --- | --- | --- | --- | --- | --- | --- | --- | --- | --- | --- | --- | --- | --- | --- | --- |
|  |  | + | + | + |  |  |  |  | + | + | + |  | + |  | 9 | 2096.0 | 0 | 0.022 |
|  |  | + | + | + |  |  |  |  | + | + | + | + | + |  | 10 | 2096.0 | 0.01 | 0.021 |
|  |  | + | + | + | + | + |  |  | + | + | + | + | + |  | 12 | 2096.0 | 0.06 | 0.021 |
|  |  | + | + |  | + |  |  |  | + | + | + |  | + |  | 9 | 2096.1 | 0.12 | 0.020 |
|  |  | + | + |  | + |  |  |  | + | + | + | + | + |  | 10 | 2096.1 | 0.16 | 0.020 |
|  |  | + | + | + | + | + |  |  | + | + | + |  | + |  | 11 | 2096.2 | 0.21 | 0.019 |
|  |  | + | + | + | + | + |  |  | + | + | + | + |  |  | 11 | 2096.5 | 0.49 | 0.017 |
|  |  | + | + | + |  | + |  |  | + | + | + |  | + |  | 10 | 2096.7 | 0.69 | 0.015 |
|  |  | + | + | + |  | + |  |  | + | + | + | + | + |  | 11 | 2096.7 | 0.69 | 0.015 |
|  |  | + | + | + | + | + |  |  | + | + | + |  |  |  | 10 | 2096.8 | 0.83 | 0.014 |
|  |  | + | + | + |  |  |  |  | + | + | + | + |  |  | 9 | 2098.8 | 0.84 | 0.014 |
|  |  | + | + |  | + |  |  |  | + | + | + | + |  |  | 9 | 2096.0 | 0.89 | 0.014 |
|  |  | + | + | + |  |  | + |  | + | + | + |  |  |  | 8 | 2097.0 | 1.00 | 0.013 |
| M  2.2 | Latency | RBS ^a)^ | Body size ratio | Body size ratio^2^ | RBS: size ratio | RBS: size ratio^2^ | Caller consis-tency | Call relia-bility | Acoustic similarity | Caller abun-dance | Grass height | Dis-tance | Wind | Group size | df | AICc | delta | weight |
|  |  | + | + | + |  |  |  |  | + |  |  | + | + |  | 9 | 4258.2 | 0 | 0.060 |
|  |  | + | + | + | + |  |  |  | + |  |  | + | + |  | 10 | 4258.9 | 0.73 | 0.041 |
|  |  | + | + | + | + | + |  |  | + |  |  | + | + |  | 11 | 4258.9 | 0.78 | 0.040 |
|  |  | + | + | + |  |  |  |  | + |  |  | + |  |  | 8 | 4259.6 | 1.48 | 0.028 |
|  |  | + | + | + |  | + |  |  | + |  |  | + | + |  | 10 | 4259.7 | 1.58 | 0.027 |
|  |  | + | + |  | + |  |  |  | + |  |  | + | + |  | 9 | 4260.0 | 1.83 | 0.024 |
|  |  | + | + | + | + |  |  |  | + |  |  | + |  |  | 9 | 4260.2 | 2.04 | 0.021 |
| M  2.3 | Duration | RBS ^a)^ | Body size ratio | Body size ratio^2^ | RBS: size ratio | RBS: size ratio^2^ | Caller consis-tency | Call relia-bility | Acoustic similarity | Caller abun-dance | Grass height | Dis-tance | Wind  speed | Group size | df | AICc | delta | weight |
|  |  | + | + | + | + | + |  |  | + | + | + |  | + |  | 12 | 4406.6 | 0 | 0.183 |
|  |  | + | + | + | + | + |  |  | + |  | + |  | + | + | 11 | 4406.6 | 0 | 0.183 |
|  |  |  | + | + |  |  |  |  | + | + | + |  | + |  | 9 | 4408.6 | 2.05 | 0.066 |
| M  2.4 | Speed of head-lift | RBS ^a)^ | Body size ratio | Body size ratio^2^ | RBS: size ratio | RBS: size ratio^2^ | Caller consis-tency | Call relia-bility | Acoustic similarity | Caller abun-dance | Grass height | Dis-tance | Wind  speed | Group size | df | AICc | delta | weight |
|  |  | + |  |  |  |  |  |  |  |  |  |  |  |  | 4 | 2194.4 | 0 | 0.191 |
|  |  |  |  |  |  |  |  |  |  |  |  |  |  |  | 3 | 2196.1 | 1.64 | 0.084 |
|  |  | + |  |  |  |  |  |  |  |  | + |  |  |  | 5 | 2196.8 | 2.39 | 0.058 |
| M  2.5 | Head-ups | RBS ^a)^ | Body size ratio | Body size ratio^2^ | RBS: size ratio | RBS: size ratio^2^ | Caller consis-tency | Call relia-bility | Acoustic similarity | Caller abun-dance | Grass height | Dis-tance | Wind  speed | Group size | df | AICc | delta | weight |
|  |  | + |  |  |  |  | + | + |  |  | + |  |  |  | 7 | 4501.9 | 0 | 0.015 |
|  |  | + |  |  |  |  | + | + |  |  | + |  | + |  | 8 | 4502.4 | 0.57 | 0.012 |
|  |  | + |  |  |  |  | + |  |  |  | + |  |  |  | 6 | 4502.5 | 0.67 | 0.011 |
|  |  | + |  |  |  |  | + | + |  |  | + | + |  |  | 8 | 4502.7 | 0.87 | 0.010 |
|  |  | + |  |  |  |  |  |  |  |  | + |  |  |  | 5 | 4502.7 | 0.88 | 0.010 |
|  |  | + |  |  |  |  | + | + | + |  | + |  |  |  | 8 | 4502.8 | 0.94 | 0.010 |
|  |  |  |  |  |  |  | + | + |  |  | + |  |  |  | 6 | 4502.8 | 0.98 | 0.009 |
|  |  | + |  |  |  |  | + |  |  |  | + |  | + |  | 7 | 4503.1 | 1.22 | 0.008 |
| M  2.6 | Scratches | RBS ^a)^ | Body size ratio | Body size ratio^2^ | RBS: size ratio | RBS: size ratio^2^ | Caller consis-tency | Call relia-bility | Acoustic similarity | Caller abun-dance | Grassheight | Dis-tance | Wind  speed | Group size | df | AICc | delta | weight |
|  |  | + | + |  | + |  |  |  |  |  | + |  |  |  | 7 | 1953.7 | 0 | 0.012 |
|  |  | + |  | + |  | + |  |  |  |  | + |  |  |  | 7 | 1953.7 | 0.06 | 0.011 |
|  |  | + |  | + |  | + |  | + |  |  | + |  |  |  | 8 | 1954.5 | 0.83 | 0.008 |
|  |  | + | + |  | + |  |  | + |  |  | + |  |  |  | 8 | 1954.7 | 0.97 | 0.007 |
|  |  | + | + |  | + |  |  |  |  |  | + |  | + |  | 8 | 1954.7 | 0.99 | 0.007 |
|  |  | + |  | + |  | + |  |  |  |  | + |  | + |  | 8 | 1954.7 | 0.99 | 0.007 |
|  |  | + | + |  | + |  |  |  |  | + | + |  |  |  | 8 | 1054.9 | 1.20 | 0.006 |

1. Receiver’s body size (RBS)
